# Hajdu–Cheney Syndrome: A Systematic Review of the Literature

**DOI:** 10.3390/ijerph17176174

**Published:** 2020-08-25

**Authors:** Jonathan Cortés-Martín, Lourdes Díaz-Rodríguez, Beatriz Piqueras-Sola, Raquel Rodríguez-Blanque, Antonio Bermejo-Fernández, Juan Carlos Sánchez-García

**Affiliations:** 1 Research Group CTS1068, Andalusia Research Plan, Junta de Andalucía, Hospital Universitario Virgen de las Nieves, 18014 Granada, Spain; jonathan.cortes.martin@gmail.com (J.C.-M.); bpiquerassola@gmail.com (B.P.-S.); 2 Research Group CTS1068, Andalusia Research Plan, Junta de Andalucía, Nursing Department, Faculty of Health Sciences, University of Granada, 18071 Granada, Spain; cldiaz@ugr.es (L.D.-R.); jsangar@ugr.es (J.C.S.-G.); 3 Andalusia Research Plan, Junta de Andalucía, Research Group CTS1068, San Cecilio Clinical University Hospital, 18071 Granada, Spain; 4 Research Group CTS 1068, Andalusia Research Plan. Junta de Andalucía, Clinical RIBER CENTER, 41018 Sevilla, Spain; antoniobermejofisio@hotmail.com

**Keywords:** Hajdu–Cheney syndrome, acroosteolysis, receptor, *NOTCH2*, connective tissue, rare diseases

## Abstract

Hajdu–Cheney syndrome (HCS) is a rare genetic disease that causes acroosteolysis and generalized osteoporosis, accompanied by a series of developmental skeletal disorders and multiple clinical and radiological manifestations. It has an autosomal dominant inheritance, although there are several sporadic non-hereditary cases. The gene that has been associated with Hajdu-Cheney syndrome is *NOTCH2*. The described phenotype and clinical signs and symptoms are many, varied, and evolve over time. As few as 50 cases of this disease, for which there is currently no curative treatment, have been reported to date. The main objective of this systematic review was to evaluate the results obtained in research regarding Hajdu–Cheney Syndrome. The findings are reported in accordance with the Preferred Reporting Items for Systematic reviews and Meta-Analyses (PRISMA) guidelines and were registered on the web PROSPERO under the registration number CRD42020164377. A bibliographic search was carried out using the online databases Orphanet, PubMed, and Scielo; articles from other open access sources were also considered. Finally, 76 articles were included, and after their analysis, we have obtained a series of hypotheses as results that will support further studies on this matter.

## 1. Introduction

### 1.1. Rationale

Hajdu–Cheney syndrome (HCS) is a rare genetic disease of the connective tissue that belongs to the osteolysis syndromes group [1]. It is registered in the OMIM (Mendelian Inheritance in Man) project database with reference #102500 and in ORPHANET under the reference ORPHA955. It is also known as acro-dento-osteo dysplasia, acroosteolysis with osteoporosis and changes in the skull and mandible, arthrodentoosteodysplasia, and serpentine fibula-polycystic kidney syndrome. The prevalence [2] of this disease is less than one person in one million (<1/1,000,000) and it is caused by a heterozygotic mutation of gene *NOTCH2* [3] located on chromosome 1p13-p11. HCS follows an autosomal-dominant inheritance pattern, although descriptions of cases with sporadic mutations can be found [4].

The disease was first described in 1948 by N. Hajdu [5] and later completed by D. Cheney in 1965 [6]. Since then, around 50 [6] cases of patients with HCS have been reported and, in general, all patients show a case of osteolysis of the distal phalanges and generalized osteoporosis, accompanied by other disorders, such as craniofacial and skeletal dysmorphia, developmental skeletal disorders, premature loss of teeth, and a short stature [7].

Due to the variability in the expression of *NOTCH2*, patients can be found with phenotypic differences between them. Furthermore, this disease presents a wide and specific clinical spectrum that is rare to encounter in full in a single patient. Therefore, reports are found of cases diagnosed with HCS presenting variable clinical manifestations that worsen over time due to their age-dependent progression, with changes from early infancy to late adulthood [8].

### 1.2. Objectives

The main objective of this systematic review was to evaluate the results obtained in research regarding Hajdu–Cheney syndrome and to provide a report that guarantees a starting point for future studies with greater complexity on this matter. The secondary objectives of this study were to investigate the different phenotypes described and to enunciate a clinical description of HCS, to obtain up-to-date information on HCS to understand the impact of this disease on affected patients, and to evaluate possible future healthcare interventions.

## 2. Materials and Methods 

### 2.1. Protocol and Registration

The method followed for this report was a systematic review of scientific literature published regarding Hajdu–Cheney syndrome in accordance with the Preferred Reporting Items for Systematic Reviews and Meta-Analyses (PRISMA) guidelines, which consist of a 27-item checklist of the most important sections of an original article, as well as a flow diagram that depicts the flow of information through the different phases of a systematic review.

This systematic review was conducted following a protocol and was registered on the web PROSPERO [9] (registration number CRD42020164377).

### 2.2. Eligibility Criteria

We selected only original articles published prior to July 2020 that provided information on HCS, without restrictions regarding the language of publication or publication date. Previous bibliographic reviews were not accepted, nor articles that were exclusively case reports.

### 2.3. Information Sources and Search

We searched the Orphanet, PubMed, and Scielo databases. We also manually searched the reference lists of included articles to find other relevant articles.

The chosen structured language was obtained from MeSH (Medical Subject Headings) terms and Health Sciences Descriptors (DeCS). The descriptors used were “Hajdu-Cheney Syndrome”, “Acro-Osteolysis”, “Receptor, *NOTCH2*”, “Connective tissue”, and “Rare diseases”, and the boolean operators used were “AND” and “OR”.

The search chains used are shown in Table 1.

### 2.4. Data Collection Process

All articles found were transferred to the web application Mendeley using the Mendeley web importer tool.

After importing all the articles to the Mendeley website, we organized them by folders according to the database from which each article had been retrieved and subsequently classified them in sub-folders according to thematic blocks. Finally, we proceeded to eliminate all duplicates, therefore achieving the definitive list for our study.

### 2.5. Study Selection

After searching the different databases, we proceeded to eliminate duplicate documents. Following the removal of duplicates, we screened all the retrieved articles by title and abstracts to identify those that fit the inclusion criteria. Next, we independently read each article, focusing on its methodology to verify whether it complied with our eligibility criteria and all articles that did not were discarded.

Three independent reviewers completed this phase of the selection process. Any disagreements were discussed with the study supervisor and resolved via a consensus-based discussion.

This study selection process is explained in more detail in the results section.

### 2.6. Data Collection Process and Data Items

We prepared a data extraction form, drew three papers one by one, modified our form after each draw, and finally developed the definitive data extraction form we used for the remaining articles. Three independent revisors extracted the data of each article. A discussion was held between the revisors to reach a consensus each time there was a disagreement on any data extracted. If the three revisors could not reach an agreement, the study supervisor was consulted.

The data items extracted included the surname of the first author, year of publication, study title, thematic block, and results. Due to the vast amount of reported studies, we divided the articles into four thematic blocks: disease genetics, description of the disease and evolution of phenotype, diagnosis and differential diagnosis, and treatment.

### 2.7. Synthesis of the Results

Considering the information this review provides, we have obtained a series of hypotheses as results that will support further, more complex studies on this matter. Alongside providing a complete analysis of this disorder, we confirmed that this syndrome, and rare diseases in general, belong to an area of medicine in which there is still much to explore, and the advancement of scientific knowledge is needed in order to progress.

## 3. Results

### 3.1. Study Selection

**Figure 1:**
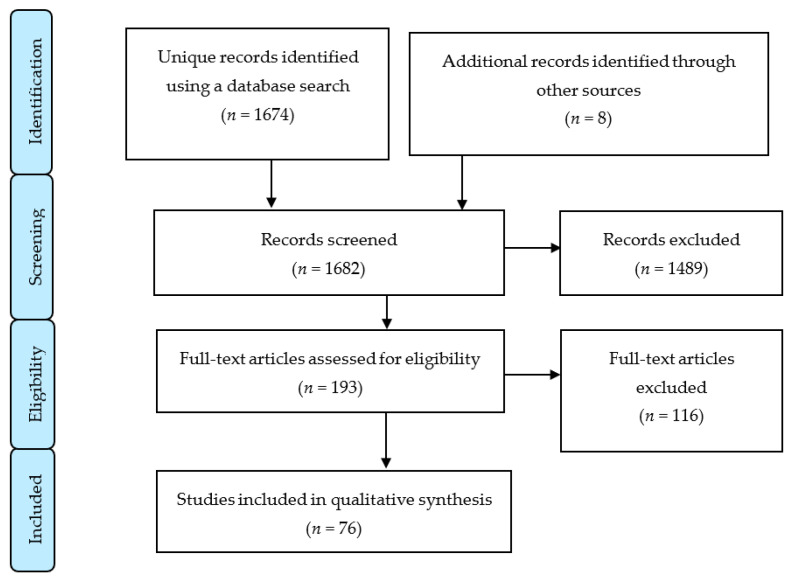
Flow diagram.

Figure 1 shows the flow diagram of the articles assessed during the production of this systematic review. 

Of the 193 articles assessed for eligibility, 117 articles were excluded, amongst which were systematic reviews, duplicate articles, and some that were exclusively case reports.

As we mentioned previously, we divided the articles into four thematic blocks: disease genetics, description of the disease and evolution of phenotype, diagnosis and differential diagnosis, and treatments.

We now present the results of the selected studies.

Table 2 shows a summary of the results of the most remarkable studies of this revision, divided by thematic block. Additionally, we include a supplementary table where the objectives, results, sample, and demographic region are shown for all 76 articles included in this systematic review (Supplementary Table S1).

### 3.2. Disease Genetics

Studies on the genetics of HCS are intimately related to the etiology of the disease, as Gibofsky [10] predicted in his study, by suspecting that the inheritance of this disease would be autosomal dominant and caused by a genetic mutation. Years later, these facts were confirmed by Simpson et al. [3] when they established that HCS is caused by a mutation on *NOTCH2*. Authors, such as Caignec [11], studied the genetics of *NOTCH* (structure, function, and signaling processes) and their relation to bone development and homeostasis in depth. They also identified mutations on *NOTCH* that are related to other disorders. Engin et al. [32] focused on the link between the *NOTCH* signaling pathway and bone development, and obtained among their results that the loss of *NOTCH* signaling correlates with an increase in osteoporosis levels. Sahlgren et al. [33] centered their projects on stem cell differentiation via the *NOTCH* signaling pathway. The study by Canalis et al. published in 2005 [13] presented the bone remodeling process involved in normal bone development, and in their 2016 study [14], they present the creation and development of a mouse model of HCS after the introduction of a mutation 6955C3T in *NOTCH2*. The heterozygotic mutant mice show shorter femurs, smaller dimensions, cancellous bones, and increased bone resorption. In 2012, Zanotti et al. [12] studied bone development in HCS, highlighting the importance of *NOTCH* in these processes and the fact that mutations on this gene lead to a loss of bone mass and acroosteolysis. In 2014, Zanotti et al. [34] further managed to inactivate *NOTCH* signaling by crossing homozygotic mice with mice where the promoter osterix governs the expression of Cre, while in 2018 [35], they presented their mouse model of HCS (*NOTCH2*tm1.1Ecan) to study the link between the disease and osteoarthritis. Vollersen et al. [36] developed a mouse model of HCS by introducing a pathogenic mutation (6272delT) on the murine gene *NOTCH2*. Changes in bone density, acroosteolysis, and an increase in the osteoblast-osteoclast index were found when compared to control cases.

### 3.3. Description of the Disease and Evolution of the Phenotype

Studying and describing the evolution of the phenotype in HCS is a highly complex task. Hajdu et al. [5] carried out the first description of the disease by presenting the case of a patient with craniofacial alterations. In his report, Cheney [6] completed the description by adding a series of clinical manifestations that accompanied cranial dysplasia. Since then, many authors have offered their contributions to this matter. Brennan et al. [8], Regev et al. [37], and Jirečková et al. [38] confirmed and justified in their studies that the disease has three main features: phenotypic variability, a wide spectrum of clinical presentation, and an age-dependent progression. Majewski et al. [39] confirmed that HCS is a hereditary disease, although sporadic cases do exist, as Descartes et al. [4] report in their study. The description of the disease is continually growing as it is directly linked to the appearance and reporting of new cases. Brown et al. [7] carry out a complete description of the disease, affirming that these patients present with low bone density and deficient bone formation. Rosenmann et al. [40], Elias et al. [41], Bruckner et al. [42], Letchumanan et al. [43], Stathopoulos et al. [44], and Nunziata et al. [16] argue that the most prevalent signs in HCS are acroosteolysis and generalized osteoporosis. Hoey et al.’s [45] study stands out as it reports a case with intrauterine fractures, as do the studies of Barakat et al. [18] and Battelino et al. [46], who remark renal alterations as their main result; Siklar’s [17] contribution regarding growth hormone and its relation to short stature in patients with HCS and Herscovici et al.’s [47] study presenting cervical instability as a sign of the disease also stand out. We must also note the studies of Bazopoulou-Kyrkanidou et al. [48] and Antoniades et al. [49], which focus on dental anomalies. Fryns’s [50] study discusses alterations in voice and speech, and Avela et al. [51] present the case of a patient with severe dural ectasia as a sign of HCS. The existence of multiple fractures, in the case of Mannstadt et al. [52], reaffirms what other authors believed on this matter. Amalnath et al. [53] mention micrognathia as another characteristic of HCS throughout his study. Another three remarkable groups in this thematic block are Takafumi et al. [54] and their case of premature ovarian failure; Sargin et al. [55], who mention congenital cardiovascular alterations; Swan et al. [56], who confirm that HCS can also cause congenital alterations of the ocular system with the results of their study.

Beyond the simple clinical manifestations, we can also find possible complications of the disease that must be included in its description. It is worth mentioning syringomyelia, as studied by Nishimura et al. [57] and Tanimoto et al. [58]. Due to thoracic deformities, breathing complications can develop, as Sasaki et al. [59] expose in their study. Ades et al. [19] and Takatani et al. [60] focus most of their studies on hydrocephalus as a complication of HCS, and the possible neurological damage is described by various authors, such as Niijima et al. [61].

There are studies on the past controversy between serpentine fibula-polycystic kidney syndrome and HCS. Several authors, such as Fryns et al. [62], Ramos et al. [15], Currarino et al. [20], Isidor et al. [21], and Gray et al. [22] published their studies to show that serpentine fibula-polycystic kidney syndrome is not an independent disease from HCS but instead another manifestation of the same disease, as both are caused by the same mutation.

### 3.4. Diagnosis and Differential Diagnosis

It is complicated to reach a definite and early diagnosis of HCS. Studies, such as Schawo’s [29], reveal that physical appearance and radiological testing will guide the diagnostic process, although the final confirmation must be genetic. Kawamura et al. [23] indicate the importance of performing magnetic resonance imaging as a complementary test to physical examination. Damian et al. [63] proposed capillaroscopy as a clinical test for HCS diagnosis.

Brennan and Pauli [8] created a diagnostic tool that establishes inclusion criteria for this syndrome based on a series of physiological parameters and genetic inheritance based on the London Dysmorphology Database. This tool facilitates the diagnosis of HCS by focusing on a series of clinical manifestations, such as acroosteolysis, wormian bones or open sutures, platybasia, premature denture loss, micrognatia, coarse face, coarse hair, midfacial flattening, short stature (<5%), and a documented positive family history. It establishes differences between adults and children due to its age-dependent progression and changes in phenotype.

For adults, three options are proposed that would lead to a positive diagnosis:

- Acroosteolysis plus three clinical manifestations, except for a documented positive family history.
- Acroosteolysis plus a documented positive family history.
- Documented positive family history plus two other manifestations, except for acroosteolysis.

For children, two options are proposed:

- Four clinical manifestations, except for a documented positive family history.
- Documented positive family history plus two manifestations.

There are studies on the differential diagnosis of HCS, as it can often resemble other diseases when trying to establish a diagnosis. Sawin et al. [26] state that basilar invagination is not a sign that is unique to HCS. Singh et al. [1] describe similarities between HCS and other acroosteolysis syndromes. In their 2011 and 2015 studies, Gripp et al. [25,64] explain that lateral meningocele, despite its clinical links with HCS, is not the same disease. O’Reilly et al. [24] point to scleroderma and sarcoidosis as disorders that also cause acroosteolysis, and therefore, the importance of including them in a correct differential diagnosis. Another remarkable study on differential diagnosis is that of Albano et al. [65] since it establishes the differences between serpentine fibula, Melnick–Needles, and HCS.

### 3.5. Treatment

In the search for an efficient treatment for HCS, there are two major aspects: pharmacological and surgical treatments.

Relevant studies on pharmacological treatments for HCS include that by Sakka et al. [31], who worked on a therapy that uses bisphosphonates and found oscillations in bone mineral density indexes of the lumbar spine. At the beginning of the study, the values decreased, then increased in response to treatment, but the effect did not persist after its interruption. Pittaway et al. [66] also studied bisphophonates but obtained different results for each patient that were dependent on age. Adami et al. [67] tried obtaining an increase in bone mineral density with denosumab but acroosteolysis persisted. Tsinopoulou et al. [68] and Al-Mayouf et al. [69] experimented with pamidronate without achieving a curative result. Hwang et al. [70] tried to slow down the process of bone degradation using zoledronic acid, and in 2007 and 2008, McKiernan et al. [30,71] carried out a pharmacological study to treat osteoporosis in HCS with an anti-resorption and anabolic therapy.

When analyzing the surgical aspect, we found studies focused on the treatment of complications of HCS, such as cervical kyphotic deformities in patients with osteoporosis by Mattei et al. [72], dental restorations by Vingerhoedt et al. [73] and Liljeström et al. [74], spinal reconstruction by de Murtagh-Schaffer et al. [28], and translation of the radius by Fujioka et al. [75] and Ornetti et al. [27], who were able to treat a vertebral compression fracture surgically. It is also worth mentioning the studies of Yamaguchi et al. [76] and August et al. [77] on anesthesia and specific treatment indications for patients with HCS prior to surgery, as well as the study on post-surgical analgesia for pain management by Zietz et al. [78].

Despite significant advances in treatment, the results of the evaluated studies on this matter indicate that a curative treatment for HCS does not yet exist.

## 4. Discussion

### 4.1. Summary of Evidence

We now present a general description of the disease.

#### 4.1.1. Epidemiology

Hajdu–Cheney syndrome has a prevalence of less than 1 in 1,000,000 live births [2]. Since 1948, approximately 50 [3] cases have been described worldwide. It is a genetic disease with autosomal-dominant inheritance [39], although sporadic cases have been reported [4].

#### 4.1.2. Etiology

As we have previously stated, HCS is a genetic disease caused by a heterozygotic mutation of *NOTCH2*. The *NOTCH* signaling pathway [11] is constituted by a series of linked occurrences that are intimately related to skeletal development and homeostasis [32]; therefore, alterations of this pathway cause disorders in both processes.

*NOTCH* receptors are transmembrane proteins [11] that have three major parts: an extracellular domain that consists of multiple EGF (epidermal growth factor)-like repeats, another intermembrane domain, and an intracellular one that consists of multiple ankyrin repeats, nuclear localization signals, and a proline-, glutamic acid-, serine-, and threonine-rich domain, known as the PEST domain, whose function is the recycling of proteins. *NOTCH* has four receptors (*NOTCH* 1, 2, 3, and 4) and five ligands (JAG1, JAG2, and DLL 1, 2, and 4).

The *NOTCH* signaling pathway activates when a cell’s ligand adheres to the receptor of the cell, provoking the separation of the intracellular domain, which travels to the nucleus of the cell where it begins to complete its function.

In HCS, there is a truncation in exon 34 of *NOTCH2* [22], which causes a protein product to be missing the PEST domain, leading to an elevated level of *NOTCH* signaling activity in multiple tissues, therefore altering the usual process. This has a noticeable impact on skeletal development and homeostasis, leading to the disease.

Several disorders have been associated with *NOTCH* mutations along with HCS [11]:

- CADASIL (cerebral autosomal dominant arteriopathy with subcortical infarcts and leukoencephalopathy): mutations of EGF-like repeats of *NOTCH3*.
- Bicuspid aortic valve: protein-truncating mutations of *NOTCH1*.
- LAL-T: protein-truncating mutations of the PEST domain of *NOTCH1*.
- Alagille syndrome: mutation of the splice acceptor of exon 33 of *NOTCH2*.

#### 4.1.3. Pathophysiology

Once a mutation has occurred, normal skeletal development is affected, causing a series of skeletal anomalies. There is a bone density deficit [7] that leads to generalized skeletal dysplasia. Osteoporosis is one of the most characteristic signs of HCS, along with acroosteolysis of distal phalanges, both of which are caused by a series of local mechanisms [16] that increase osteoclastic activity and impact bone formation negatively. Congenital defects in ossification can be found in fetal cartilage [7], causing peripheral dysostosis that worsens the acroosteolysis.

Understanding these processes helps us to comprehend some of the clinical manifestations that are seen in the wide spectrum of clinical presentations this syndrome provides: fractures in long bones due to bone demineralization [52], frequent respiratory infections caused by thoracic deformities and ventilatory restriction [24], basilar invagination [19] and its neurological alterations, and short stature due to vertebral collapse. These are some of the clinical complications that arise from these processes that, when considered alongside the age-dependent progression of HCS, make this syndrome so complex.

#### 4.1.4. Clinical Manifestations

This syndrome has three major characteristics regarding symptoms: a variable phenotype [37], a wide spectrum of clinical presentation [8], and an age-dependent progression [38]. Following these three parameters, some patients are diagnosed with HCS with different clinical presentations and phenotypic differences person to person, and these manifestations tend to evolve over time. The following clinical manifestations are the most representative of HCS:

- Cranial alterations: bathrocephaly, presence of multiple wormian bones, delayed suture closure, thickened dome of the skull, absent frontal sinuses, elongated sella turcica, small jaw, basilar invagination, dolichocephaly, and occipital prominence.
- Facial alterations: coarse and dysmorphic facies, elongated philtrum, micrognathia, low-set ears, telecanthus, sinofridia, bushy eyebrows, long eyelashes, wide nose, high arched palate, premature denture loss, jaw malocclusion, hirsutism, and hypertelorism.
- Musculoskeletal alterations: short stature, short neck, fractures of long bones, joint laxity, biconcave vertebrae, kyphoscoliosis, cervical instability, vertebral collapse, genu valgum, serpentine fibula, acroosteolysis, pseudoclubbing, short fingers, Hippocratic fingers, progressive distal bone resorption, bone demineralization, osteopenia, and osteoporosis.
- Cardiovascular alterations: congenital heart disease, patent arterial duct, and sept defects.
- Digestive alterations: intestinal malrotation.
- Neurological alterations: hydrocephalus and lateral meningocele.
- Renal alterations: hypospadias, cryptorchidism, renal cysts, and kidney failure.
- Respiratory alterations: thoracic deformities, ventilatory restriction, and recurrent infections.
- Other alterations: delayed motor development, hearing loss, changes of the voice, deep voice, short nails, plantar ulcers, and hernias.

**Figure 2:**
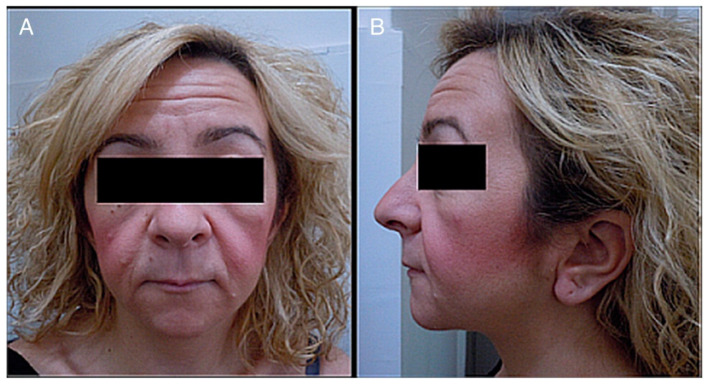
(**A**) Photograph of a patient’s face. (**B**) Lateral photograph of a patient’s head and face. The following are noted: small face, telecanthus and downslated palpebral fissures, micrognathia, small mouth, thin lips, long philtrum and full cheeks, low-positioned ears with a crease in the lobules, short neck, and coarse hair.

**Figure 3:**
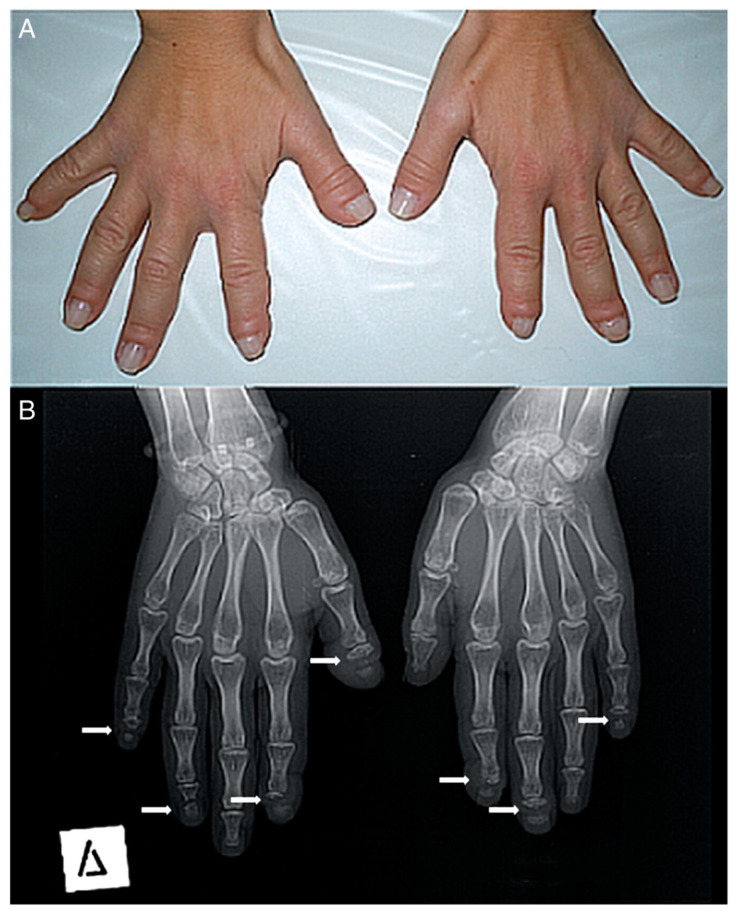
(**A**) Photograph of a patient’s hands. Many of her fingers are thick (predominately the right thumb) with pseudo-clubbing. (**B**) Anteroposterior radiograph of a patient’s hands. Osteolysis of the distal phalanges is found in most of the fingers (only the left thumb, the left fourth, and the right third fingers have a normal appearance). In all lesions, osteolysis has a transverse pattern across the width of the terminal phalanx (white arrows).

**Figure 4:**
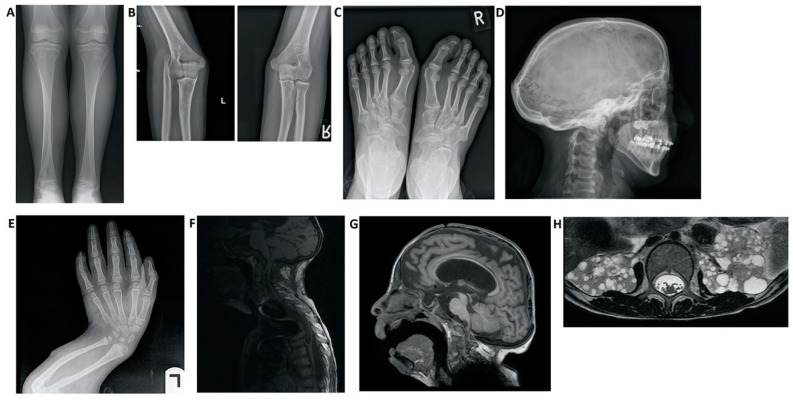
Imaging findings in patients with Hajdu–Cheney syndrome. (**A**) Radiograph of the frontal view of the tibia and fibula. Note the elongated and medially deviated fibula, superposing the tibia bone, referred to as a “serpentine fibula”. (**B**) Radiographs of the lateral view of the left arm showing dislocation of the radial head. (**C**) Radiograph of the frontal view of the feet showing hallux valgus, crowded metatarsal bones, short foot distal digits, and acroosteolysis signs at distal phalanges of the first digit. (**D**) Radiograph of the lateral view of the skull, demonstrating batrocephaly, frontal sinuses hypoplasia, and multiple occipital wormian bones. (**E**) Radiograph of the frontal view of the hands, showing crowded metacarpal bones, short hand distal digits, and acroosteolysis signs at the distal phalanges. (**E**) Radiograph of the lateral view of the arm, showing shortening of the distal fingers and long bones of the arm, and a curved radius and ulna. (**F**) Spinal MRI showing deformations of the cervical spine, as well as a significant syrinx of the cord. (**G**) Brain MRI. Note the ventricular enlargement with a VP (ventriculo-peritoneal) shunt, frontal hypoplasia of the sinuses, and a small foramen magnum. (**H**) Abdominal MRI showing multiple bilateral kidney cysts.

**Figure 5:**
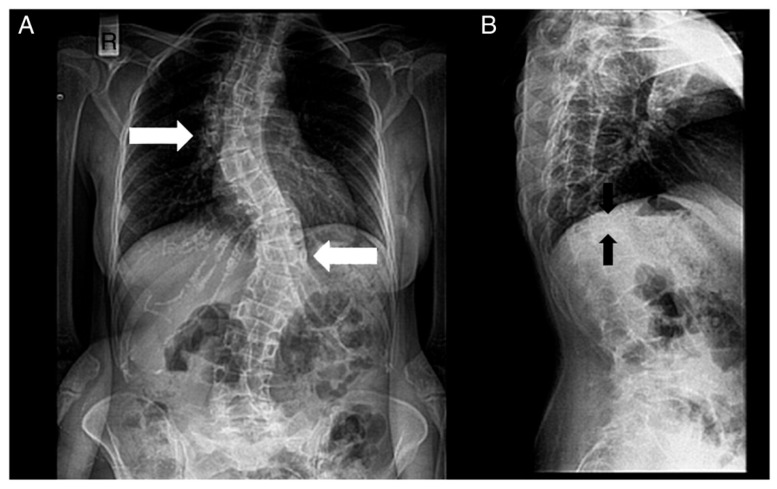
Radiographs of a patient’s spine. (**A**) Anteroposterior view. (**B**) Lateral view. The following are noted: scoliosis (double major—with a right thoracic and a left thoracolumbar curve—white arrows), and biconcave deformities of the upper and lower endplate (fishbone deformity—black arrows) of many vertebrae and decreased bone density.

The most relevant clinical manifestations are shown in Figure 2, Figure 3, Figure 4 and Figure 5.

The most frequent clinical complications in this syndrome are basilar invagination, and consequently, brain damage, hydrocephalus [19], syringomyelia [57], vertebral collapse due to compression [27], and ventilatory restriction caused by a thoracic deformity [24].

There is a subgroup of patients within this syndrome that present two distinctive signs: serpentine fibula and polycystic kidneys. Initially, it was thought that patients with these signs belonged to a separate disease [65], different from HCS, but genetic studies demonstrated both originate from the same mutated gene [15]. The variable expression of *NOTCH2* justifies the frequent association of serpentine fibula and polycystic kidneys as nothing but another manifestation of HCS and not an independent syndrome [22].

Based on the age-dependent progression of this syndrome, it has been shown that after monitoring several cases over time, the phenotypes and symptoms gradually worsen. There are a series of stages of the disease according to age, allowing for seven generational divisions [8]:

- birth (<1 year old)
- early childhood (ages 1–5)
- childhood (ages 6–12)
- adolescence (ages 13–19)
- early adulthood (ages 20–33)
- middle adulthood (ages 36–65)
- late adulthood (65+).

We highlight the importance of presenting an updated report of the variability of manifestations and the changing phenotype of this disease.

#### 4.1.5. Diagnosis

The diagnosis of HCS is suspected via the observation of physical appearance and radiological findings [29], but the final diagnosis is reached via genetic sequencing of exon 34 of *NOTCH2*.

Brennan and Pauli [8] created a diagnostic tool that establishes the inclusion criteria for this syndrome.

It is worth highlighting the need for establishing a differential diagnosis with other disorders and syndromes that share clinical manifestations and could generate diagnostic uncertainty.

Regarding its osteolytic nature [1], HCS can be compared to some of the disorders that belong to the group of osteolysis syndromes, such as Torg, François, Whyte–Hemingway, Winchester, and a new syndrome known as Talo-patello-scaphoid osteolysis, synovitis, and short fourth metacarpals. One of the main characteristics of the disease to be analyzed is acroosteolysis but we can also find other disorders that present as signs, such as scleroderma, sarcoidosis, neuropathic disorders, and rheumatoid syndromes [24]. Progeria and pycnodysostosis are another type of disorder that cause congenital acroosteolysis. Including Paget’s disease or other osteoporosis syndromes in the differential diagnosis is also of interest due to their osteoporotic nature. There are studies on the differential diagnosis between HCS and lateral meningocele considering phenotypic similarities [25,64] and with Alagille syndrome because of their genetic links. Other syndromes that share basilar invagination [26] amongst their clinical manifestations are osteogenesis imperfecta, congenital osteochondrodysplasia, and spondyloepiphyseal dysplasia, and could also be considered when creating the differential diagnosis for HCS.

#### 4.1.6. Treatment

There is no definitive or effective pharmacological treatment for HCS at present, and although certain trials with bisphosphonates have been developed [30], there is insufficient evidence of their effectiveness. Surgical intervention as a method to avoid complications has proven to be effective in certain cases [28]. The current treatment for HCS is based on the management of complications and underlying problems in order to improve the patient’s quality of life and life expectancy. Certain studies consider the manipulation of gamma-secretase inhibitors as a possible way to prevent this disorder [11].

#### 4.1.7. Prognosis

HCS is classified as a rare genetic disease but there are no studies that offer a global perspective on the prognosis and quality of life of affected patients. The severity of the disease depends on the affected organs, clinical complications, and the degenerative evolution of each patient. The generalized osteoporosis and the development of acroosteolysis will cause fractures, difficulty with walking, and dependency for everyday life activities.

The prognosis worsens when complications such as basilar invagination exist, causing neurologic alterations, or thoracic deformities that cause ventilatory restriction. Due to the low prevalence and the lack of qualitative information about this syndrome, it is difficult to know the burden of disease and the years of healthy life lost. Researchers should discuss the results and how they can be interpreted given previous studies and the working hypotheses. The findings and their implications should be discussed in the broadest context possible. Future research directions may also be highlighted.

### 4.2. Limitations

Despite the fact that undeniable progress has been made by the scientific community regarding this syndrome at present (its genetic origin is known, there is a general understanding of the processes that lead to disease development, and that there is ongoing research to further understand this disorder), the management of HCS is still based on the treatment of the different clinical complications that arise and on the evaluation of the individual needs of each patient to provide care that improves their quality of life.

Due to the low prevalence of HCS and the number of reported cases, obtaining a complete description and global perspective of the disorder is complex. The geographical dispersion of the reported cases, the time between them, a lack of constant monitoring of identified cases, and the absence of specific descriptive protocols for this disorder are some of the obstacles that hinder the development of research into this syndrome.

An early and definitive diagnosis of HCS is not an easy task, mainly due to the vast amount of clinical manifestations of the disease, phenotypic differences between cases, and the evolution of manifestations over time. When the wide range of disorders and syndromes with overlapping clinical signs and symptoms that lead to diagnostic uncertainty is also considered, the diagnosis of HCS proves challenging.

### 4.3. Possible Future Lines of Research

As in all rare diseases without treatment, the best option is to improve patients’ quality of life as much as possible. The establishment of a specific multidisciplinary intervention plan would be of great help for the everyday lives of these patients. To do so, it would be essential to:

- Revise all the cases described in the literature and describe new cases to obtain a large and reliable sample of patients with HCS with whom a descriptive study could be carried out. Such a study would serve not only to identify the cases of this disorder and study its prevalence but also to analyze the complete phenotype of HCS in depth, allowing for a greater understanding of this syndrome and contribute to an earlier diagnosis in new cases.
- Standardize protocols for the evaluation of signs and symptoms, diagnostic orientation, and disease management. Action protocols and specific intervention plans are basic and necessary tools for the universalization of care for patients with HCS. The use of a nursing methodology and its taxonomy NANDA (North American Nursing Diagnosis Association) - NIC (Nursing Interventions Classification) - NOC (Nursing Outcomes Classification) would provide a universal, individualized, and multidisciplinary approach to this disorder.
- Perform a qualitative study on HCS to understand the impact on the quality of life and daily activities. Such a study would aim to report on the level of dependency and adaptation of these patients and evaluate possible future healthcare interventions.

## 5. Conclusions

Considering the scientific and clinical variance of HCS, there is a need for standardization and universalization of the evaluation and diagnostic testing to simplify and facilitate progress in the research setting. A detailed description of cases would improve the diagnosis timing, quality of treatment, and overall assistance of each patient.

## Figures and Tables

**Table 1:** Search chains used.

Information Source	Search Chain
ORPHANET	Código ORPHA: 955
PUBMED	((((Hajdu-Cheney Syndrome) OR (Acro-Osteolysis)) OR (Receptor, *NOTCH2*)) OR (Connective tissue)) OR (Rare diseases) (((((((“hajdu-cheney syndrome”[MeSH Terms] OR (“hajdu cheney”[All Fields] AND “syndrome”[All Fields])) OR “hajdu cheney syndrome”[All Fields]) OR ((“hajdu”[All Fields] AND “cheney”[All Fields]) AND “syndrome”[All Fields])) OR “hajdu cheney syndrome”[All Fields]) OR (((“acro-osteolysis”[MeSH Terms] OR “acro osteolysis”[All Fields]) OR (“acro”[All Fields] AND “osteolysis”[All Fields])) OR “acro osteolysis”[All Fields])) OR ((((“receptor, *NOTCH2*”[MeSH Terms] OR (“receptor”[All Fields] AND “*NOTCH2*”[All Fields])) OR “*NOTCH2* receptor”[All Fields]) OR (“receptor”[All Fields] AND “*NOTCH2*”[All Fields])) OR “receptor *NOTCH2*”[All Fields])) OR ((“connective tissue”[MeSH Terms] OR (“connective”[All Fields] AND “tissue”[All Fields])) OR “connective tissue”[All Fields])) OR ((“rare diseases”[MeSH Terms] OR (“rare”[All Fields] AND “diseases”[All Fields])) OR “rare diseases”[All Fields])
SCIELO	(Hajdu-Cheney Syndrome) OR (Acro-Osteolysis) OR (Receptor, *NOTCH2*)

**Table 2:** Summary of the results of the most remarkable studies of this revision, divided by thematic block.

Authors	Article	Thematic Block	Results
Gibofsky [10] (1987)	Genetics of the Hajdu–Cheney Syndrome	Disease genetics	The existing association between *NOTCH* and Hajdu–Cheney syndrome (HCS), focusing on the signaling pathway and other disorders caused by *NOTCH* mutations.
Le Caignec [11] (2011)	Pathologies humaines et récepteurs *NOTCH*		
Zanotti et al. [12] (2012)	*NOTCH* regulation of bone development and remodeling and related skeletal disorders.		
Canalis et al. [13] (2005)	The fate of circulating osteoblasts		
Canalis et al. [14] (2016)	Hajdu Cheney mouse mutants exhibit osteopenia, increased osteoclastogenesis, and bone resorption		
Ramos et al. [15] (1998)	Further evidence that the Hajdu-Cheney Syndrome and the ‘‘Serpentine Fibula-Polycystic Kidney Syndrome’’ are a single entity	Description of the disease and evolution of the phenotype	Descriptions of the clinical manifestations of the disease, highlighting its variable phenotype, the wide spectrum of clinical presentation, and the age-dependent progression and possible complications. Resolution of the controversy between serpentine fibula-polycystic kidney syndrome and HCS, proving it is only another manifestation of HCS, not an independent disorder as was previously believed.
Nunziata et al. [16] (1990)	High turnover osteoporosis in acro-osteolysis (Hajdu-Cheney Syndrome)		
Brown et al. [7] (1976)	The acro-osteolysis syndrome: morphologic and biochemical studies		
Brennan et al. [8] (2001)	Hajdu-Cheney Syndrome: evolution of phenotype and clinical problems		
Siklar et al. [17] (2000)	Hajdu-Cheney Syndrome with growth hormone deficiency and neuropathy		
Barakat et al. [18] (1996)	Kidney abnormalities in Hajdu-Cheney Syndrome		
Ades et al. [19] (1993)	Hydrocephalus in Hajdu-Cheney Syndrome		
Currarino [20] (2009)	Hajdu-Cheney Syndrome associated with serpentine fibulae and polycystic kidney disease		
Isidor et al. [21] (2011)	Truncating mutations in the last exon of *NOTCH2* cause a rare skeletal disorder with osteoporosis		
Gray et al. [22] (2012)	Serpentine fibula polycystic kidney syndrome is part of the phenotypic spectrum of Hajdu–Cheney Syndrome		
Kawamura et al. [23] (1991)	Hajdu-Cheney Syndrome: MR imaging	Diagnosis and differential diagnosis	We establish the necessary conditions for a diagnostic orientation toward HCS and propose several related disorders that are useful for a differential diagnosis.
O’Reilly et al. [24] (1994)	Hajdu-Cheney Syndrome		
Singh et al. [1] (2003)	Talo-patello-scaphoid osteolysis, synovitis, and short fourth metacarpals in sisters: A new syndrome?		
Gripp et al. [25] (2011)	Lateral meningocele syndrome and Hajdu–Cheney Syndrome: different disorders with overlapping phenotypes		
Sawin et al. [26] (1997)	Basilar invagination in osteogenesis imperfecta and related osteochondrodysplasias: medical and surgical management		
Ornetti et al. [27] (2012)	Osteoporotic compression fracture revealing Hajdu-Cheney Syndrome	Treatment	There is no curative treatment. There are several studies on bisphosphonates, although there is no clear evidence of their effectiveness. Surgical intervention to prevent complications is effective in certain cases. The current treatment of HCS is focused on the management of complications and underlying problems to improve the quality of life and life expectancy.
Murtagh-Schaffer et al. [28] (2008)	Spinal reconstruction in Hajdu-Cheney Syndrome		
Schawo et al. [29] (2006)	Junge frau mit rückenschmerzen und akroosteolysen		
McKiernan et al. [30] (2007)	Integrated anti-remodeling and anabolic therapy for the osteoporosis of Hajdu–Cheney Syndrome		
Sakka et al. [31] (2017)	Bone structural characteristics and response to bisphosphonate treatment in children with Hajdu-Cheney Syndrome		

## Supplementary Materials

The following are available online at https://www.mdpi.com/1660-4601/17/17/6174/s1, Table S1: Objectives, results, sample and demographic region shown for all 76 articles.
