# Supplementary material for: Hajdu–Cheney Syndrome: A Systematic Review of the Literature
Source: Int J Environ Res Public Health. 2020 Aug 25;17(17):6174. doi: 10.3390/ijerph17176174 (PMC7504254; doi:10.3390/ijerph17176174)
Supplement: Supplementary file 1 [file ijerph-17-06174-s001.zip › ijerph-895152-supplementary.docx]

| AUTHOR | ARTICLE | OBJETIVES | RESULTS | SAMPLE | DEMOGRAPHIC REGION |
| --- | --- | --- | --- | --- | --- |
| *Disease Genetics* | | | | | |
| Gibofsky (9)(1987) | Genetics of the Hajdu‐Cheney syndrome. | To present basic aspects of HCS genetics. | Theoretical approach to the origins of HCS. Initial evidence of HCS having autosomal dominant inheritance. | - | New York  (United States of America) |
| Canalis et al.(12) (2005) | The fate of circulating osteoblasts. | To present processes involved in normal bone physiology. | Complete description of the bone remodelling process. | - | Connecticut  (United States of America) |
| Sahlgren et al.(32) (2006) | *NOTCH* signaling and its integration with other signaling mechanisms. | To study stem cell differentiation via the *NOTCH* signalling pathway. | Highlights the importance of *NOTCH* in the destination of steam cells, cellular differentiation in several organs and its integration with other signalling pathways. | - | Stockholm  (Sweden) |
| Engin et al. (31)(2008) | Dimorphic effects of *NOTCH* signaling in bone homeostasis. | To study the link between the *NOTCH* signalling pathway and abnormal bone development. | The specific increase in osteoblast with *NOTCH* function causes severe osteosclerosis due to a higher proliferation of immature osteoblasts. However, the loss of all the *NOTCH* signalling in osteoblasts is associated to osteoporosis. | - | Houston  (United States of America) |
| Simpson et al.(3) (2011) | Mutations in *NOTCH*2 cause Hajdu-Cheney syndrome, a disorder of severe and progressive bone loss. | To understand the genetic origin of HCS. | Identification of mutations on *NOTCH*2 that cause HCS. | 14 | London  (United Kingdom) |
| Caignec(10)  (2011) | Pathologies humaines et récepteurs *NOTCH*. | To study the *NOTCH* signalling pathway. | Development of the genetics of *NOTCH* (structure, function and signalling processes) and to identification of *NOTCH* mutations and their respective diseases. | - | Nantes  (France) |
| Zanotti et al. (11)(2012) | *NOTCH* regulation of bone development and remodeling and related skeletal disorders. | To study abnormal bone development in HCS. | *NOTCH* is key in the regulation of bone development. *NOTCH* mutations have a negative impact causing bone loss and acroosteolysis, | - | Connecticut  (United States of America) |
| Zanotti et al.(33) (2014) | *NOTCH*1 and *NOTCH*2 expression in osteoblast precursors regulates femoral microarchitecture. | To study the expression of *NOTCH* in osteoblast in mouse models. | They crossed homozygotic mice with mice where the promoter *osterix* governs the expression of *Cre* to inactivate *NOTCH* signalling and analyse bone parameters. | - | Connecticut  (United States of America) |
| Canalis et al. (13)(2016) | Hajdu-Cheney mouse mutants exhibit osteopenia, increased osteoclastogenesis, and bone resorption. | Presentation of the mouse model of HCS. | Creation and development of a HCS mouse model by introducing a mutation 6955C3T on *NOTCH*2. | - | Connecticut  (United States of America) |
| Zanotti et al. (34)(2018) | Mice harboring a Hajdu Cheney Syndrome mutation are sensitized to osteoarthritis. | To study the link between HCS and osteoarthritis in the mouse model. | The mouse model of the disease (*NOTCH*2tm1.1Ecan) confirmed its sensitivity to development of osteoarthritis in unstable joints. | - | Connecticut  (United States of America) |
| Vollersen et al. (35) (2018) | High Bone Turnover in Mice Carrying a Pathogenic *NOTCH*2 Mutation Causing Hajdu-Cheney Syndrome. | To analyse the role of *NOTCH*2 in bone remodelling. | Development of a mouse model for HCS by introducing a pathogenic mutation (6272delT) in the murine gene *NOTCH*2. Changes in bone density, acroosteolysis and an increase in the osteoblast-osteoclast index were found when compared to control cases. | - | Hamburg  (Germany) |
| *Description of the disease and evolution of phenotype* | | | | | |
| Hajdu et al. (5)(1948) | Cranio-skeletal dysplasia. | Presentation of a new syndrome | First description and definition of HCS. | 1 | Prague  (Czech Republic) |
| Cheney W. (6)(1965) | Acro-Osteolysis. | To develop and define HCS as a disease. | Complete definition of HCS. | 5 | Michigan  (United States of America) |
| Brown et al.(7) (1976) | The acro-osteolysis syndrome: Morphologic and biochemical studies. | To study HCS at a clinical and biochemical level. | Description of the clinical presentation of HCS. Presentation, description and comparison of two new cases with those previously described at a clinical and biochemical level. | 2 | Minneapolis  (United States of America) |
| Elias et al.(40) (1978) | Hereditary osteodysplasia with acro-osteolysis (the Hajdu-Cheney syndrome). | Presentation of two cases of HCS | Description of two cases where a bone biopsy o fan affected area was performed that showed that a neurovascular dysfunction and local liberation of osteolytic mediators may be involved in the pathogenics of this disorder. | 2 | Siracusa  (Italy) |
| Rosenmann et al.(39) (1979) | Sporadic idiopathic acro-osteolysis with cranio-skeletal dysplasia, polycystic kidneys and glomerulonephritis A case of the Hajdu-Cheney syndrome. | Presentation of a case of HCS. | Description of a case of HCS with acroosteolysis, craniofacial alterations and renal alterations. | 1 | Jerusalem  (Israel) |
| Hoey et al. (44)(1983) | Hajdu-Cheney syndrome associated with intrauterine fractures and arachnoid cysts | Presentation of a case of HCS. | Description of a case of HCS, highlighting a cranial deformity due to multiple fractures prior to birth. | 1 | London  (United Kingdom) |
| Niijima et al. (60)(1984) | Familial osteodysplasia associated with trigeminal neuralgia: Case report. | Presentation of a case of HCS. | Description of the first case of HCS that presents with trigeminal neuralgia requiring surgical treatment. | 1 | Kyoto  (Japan) |
| Herscovici et al.(46) (1990) | Cervical instability as an unusual manifestation of Hajdu-Cheney syndrome of acroosteolysis. | Presentation of a case of HCS. | Description of a case of HCS with skeletal alterations that include cervical instability. | 1 | London  (United Kingdom) |
| Nunziata et al.(15) (1990) | High turnover osteoporosis in acro-osteolysis (Hajdu-Cheney syndrome). | To study the existing link between physiopathology and phenotype in HCS. | Confirmation regarding acroosteolysis and osteoporosis caused by a series of localized action mechanisms stimulating osteoclastic resorption. Report of one case. | 1 | Naples  (Italy) |
| Ades et al. (18)(1993) | Hydrocephalus in Hajdu-Cheney syndrome. | To inform of possible complications of HCS. | Presentation, through the description of a case, of one of the possible complications of HCS: basilar invagination. | 1 | Adelaide  (Australia) |
| Barakat et al. (17)(1996) | Kidney abnormalities in Hajdu-Cheney syndrome | To analyse kidney alterations in HCS. | The importance of reviewing kidney diseases to establish a complete diagnosis of patients with HCS. Renal insufficiency, vesicoureteral reflux, cystic disease, glomerulonephritis, chronic kidney disease and hypertension may be warning signs during childhood for early diagnosis. Presentation of a case. | 1 | Saudi Arabia |
| Tanimoto et al. (57)(1996) | Syringomyelia associated with Hajdu-Cheney syndrome: Case report. | Presentation of a case of HCS. | Description of a case of HCS with neurological alterations caused by syringomyelia requiring surgical treatment. | 1 | Kobe  (Japan) |
| Nishimura et al.(56) (1996) | Syringohydromyelia in Hajdu-Cheney syndrome | Presentation of a case of HCS. | Description of a case where syringomyelia appears as a complication of HCS. | 1 | Tokyo  (Japan) |
| Fryns (61)(1997) | Serpentine fibula syndrome: a variant clinical presentation of Hajdu-Cheney syndrome? | Analysis of the controversy between HCS and SFPCK. | First notions that both syndromes may actually be the same clinical entity. | - | Leuven  (Belgium) |
| Sawin et al.(25) (1997) | Basilar invagination in osteogenesis imperfecta and related osteochondrodysplasias: Medical and surgical management. | To perform a study on basilar invagination as a clinical complication of diseases belonging to osteogenesis imperfecta group. | Analysis of basilar invagination as a complication of certain bone diseases, the possible treatments and response to them before, during and after. The study is carried out with a sample of 25 cases, of which 4 have HCS. | 25 | Iowa  (United States of America) |
| Ramos et al. (1998)(14) | Further evidence that the Hajdu-Cheney syndrome and the serpentine fibula-polycystic kidney syndrome are a single entity | To establish that HCS and SFPCK (Serpentine fibula-polycystic kidney syndrome) constitute the same syndrome. | Revision and comparison of the cases describe in the literature both for HCS and SFPCK. Confirmation that both syndromes belong to the same entity. | 55 | Zaragoza  (Spain) |
| Leidig-Bruckner et al. (41)(1999) | Severe osteoporosis in familial Hajdu-Cheney syndrome: Progression of acro-osteolysis and osteoporosis during long-term follow-up. | Analysis of the evolution of HCS over time in two patients. | HCS is a degenerative disease. The clinical finding found in both cases evolve negatively with the passing of time. | 2 | Heidelberg  (Germany) |
| Siklar et al. (16)(2000) | Hajdu-Cheney syndrome with growth hormone deficiency and neuropathy | To study the existing link between short stature and neurophysiological problems with the HCS phenotype. | Inclusion in the HCS phenotype of the deficit of growth hormone and the presence of peripheral motor neuropathies. Presentation of a case. | 1 | Ankara  (Turkey) |
| Brennan et al. (8)(2001) | Hajdu–Cheney syndrome: evolution of phenotype and clinical problems. | To establish a complete definition of HCS phenotype. | HCS presents a variable phenotype, a wide spectrum of clinical presentation and age-dependent progression. Tool for the inclusive diagnosis of HCS. | 6 | Wisconsin  (United States of America) |
| Antoniades et al.(48) (2003) | Hajdu-Cheney syndrome (acro-osteolysis): A case report of dental interest. | Presentation of a case of HCS. | Description of a case of HCS that required surgical treatment of the oral cavity due to skeletal alterations. | 1 | Tesalonica  (Grece) |
| Bazopoulou-Kyrkanidou et al.(47) (2007) | Periodontitis associated with Hajdu-Cheney Syndrome | Presentation of a case of HCS. | Description of a case of HCS with alterations of the oral cavity and premature denture loss, a sign related to HCS phenotype. | 1 | Athens  (Grece) |
| Fryns et al. (49)(2008) | Vocal cord paralysis and cystic kidney disease in Hajdu-Cheney syndrome | Presentation of a case of HCS. | Description of a case of HCS with respiratory alterations and vocal cord paralysis. | 1 | Leuven  (Belgium) |
| Letchumanan et al.(42) (2009) | A patient with progressive shortening of the fingers. | Presentation of a case of HCS. | Description of a case that highlights acroosteolysis as a significant signo f HCS phenotype. | 1 | Malasya |
| Currarino(19)  (2009) | Hajdu-Cheney syndrome associated with serpentine fibulae and polycystic kidney disease | To study the existing link between HCS and SFPCK. | Revision and comparison of the cases of HCS and SFPCK described to date. Presentation of a case. Inclusion of new signs in the HCS phenotype. | 1 | Dallas  (United States of America) |
| Isidor et al. (20)(2011) | Truncating mutations in the last exon of *NOTCH*2 cause a rare skeletal disorder with osteoporosis | Analysis of the link between HCS, SFPCK, Alagille syndrome and Melnick Needles ay a genetic and clinical level. | Confirmation that the mutations that cause these disorders belong to different genes. Presentation of a case. Comparison of two cases of HCS with another of the different syndromes. | 1 | Nantes  (France) |
| Majewski et al.(38) (2011) | Mutations in *NOTCH*2 in families with Hajdu-Cheney syndrome | To study the origin of HCS. | Genetic sequencing of the sample and the obtained results confirm that HCS is caused by a mutation on *NOTCH*2. | Cohort 7 families | Quebec  (Canada) |
| Avela et al. (50)(2011) | Hajdu-Cheney syndrome with severe dural ectasia. | Presentation of a case of HCS. | Description and evolution of a case of HCS with severe dural ectasia | 1 | Helsinki  (Finland) |
| Nozaki et al.(53) (2012) | A girl with Hajdu-Cheney syndrome and premature ovarian failure. | Presentation of a case of HCS. | Description of a case of HCS that presents premature ovarian failure as a possible complication of the disease. | 1 | Fukuoka  (Japan) |
| Gray et al. (21)(2012) | Serpentine fibula polycystic kidney syndrome is part of the phenotypic spectrum of Hajdu-Cheney syndrome. | To study the controversy between SFPCK and HCS | Insists that SFPCK simply another sign of HCS. Both answer to the same mutation of *NOTCH*2. Presentation of two cases. | 2 | Dunedin  (New Zeland) |
| Stathopoulos et al. (43)(2013) | Severe osteoporosis and mutation in *NOTCH*2 gene in a woman with Hajdu-Cheney syndrome | Presentation of a case of HCS. | Description of a case of HCS that presents most of the clinical and radiological findings. The genetic diagnosis is established, confirming the link between HCS and *NOTCH*2. | 1 | Athens  (Grece) |
| Sargin et al.(54) (2013) | Hajdu-Cheney syndrome with ventricular septal defect. | Presentation of a case of HCS. | Description of a case of HCS with cardiac alterations and plantar ulcers. | 1 | Aydin  (Turkey) |
| Mannstadt et al. (51)(2014) | A 27-year-old man with severe osteoporosis and multiple bone fractures. | Presentation of a case of HCS. | Description of a case that presents fractures and osteoporosis but etiology is unknown. | 1 | Boston  (United States of America) |
| Descartes et al.(4) (2014) | Hajdu-Cheney syndrome: Phenotypical progression with de-novo *NOTCH*2 mutation. | To study the phenotype of HCS. | Description of a clinical case. Confirmation that the HCS phenotype has an age-dependant progression. | 1 | Birmingham  (United Kingdom) |
| Battelino et al.(45) (2016) | End-Stage Renal Disease in an Infant With Hajdu-Cheney Syndrome. | Presentation of a case of HCS. | Description of a case of HCS with kidney abnormalities. First case of HCS with renal failure during childhood. | 1 | Liubliana  (Slovenia) |
| Deepak et al.(52) (2016) | Hajdu-Cheney syndrome - A rare cause of micrognathia. | Presentation of a case of HCS. | Description of a case of HCS with evident micrognatia and acroosteolysis. | 1 | Puducherry  (India) |
| Jirečková et al.(37) (2018) | The Age Dependent Progression of Hajdu-Cheney Syndrome in Two Families. | To analyse the evolution of phenotype and clinical presentation of HCS according to age. | Description of two cases of HCS from two different families. Highlights one of the most remarkable characteristics of HCS: age-dependant progression. | 2 | Prague  (Czech Republic) |
| Swan et al.(55) (2018) | Congenital Glaucoma: a Novel Ocular Manifestation of Hajdu-Cheney Syndrome. | Presentation of a case of HCS. | Description of a case of HCS with visual alterations. First case where congenital glaucoma is presented as a sign. | 1 | Brisbane  (Australia) |
| Sasaki et al. (58)(2019) | Fatal case of Hajdu-Cheney syndrome with idiopathic pulmonary hemosiderosis | Presentation of a case of HCS. | Description of a case of HCS with respiratory alterations and a remarkable sign of idiopathic pulmonary hemosiderosis. | 1 | Hachinohe  (Japan) |
| Takatani et al. (59)(2019) | Hajdu-Cheney syndrome: Infantile onset of hydrocephalus and serpentine fibulae | Presentation of a case of HCS. | Description of a case of HCS that present from early age with hydrocephalus and platybasia, clinical finding identified by MRI. Another remarkable sign of this case is serpentine fibula, reinforcing the link between SF and HCS. | 1 | Chiba  (Japan) |
| Regev et al. (36)(2019) | Phenotype variability in Hajdu-Cheney syndrome. | To study the phenotype variability through two cases. | Description of two cases of HCS with two different phenotypes. Confirmation that HCS presents a variable expression of the same mutated gene. | 2 | Tel-Hashomer  (Israel) |
| *Diagnosis and Differential Diagnosis* | | | | | |
| Kawamura et al.(22) (1991) | Hajdu-Cheney Syndrome: MR imaging. Neuroradiology. | To carry out the surveillance of a previously describe case of HCS 10 years after by using MRI as a diagnostic tool. | Revision of a previously described case. Evaluation of new radiological findings detected by MRI. Confirmation that the HCS phenotype presents an age-dependant progression. | 1 | Tenri  (Japan) |
| O’Reilly et al.(23) (1994) | Hajdu-Cheney syndrome. | To establish a differential diagnosis for HCS. | Presentation of a case focusing on the differential diagnosis of HCS with other disorders such as sclerodermia, sarcoidosis and progeria, among others. | 1 | London  (United Kingdom) |
| Singh et al. (1)(2003) | Talo-patello-scaphoid osteolysis, synovitis, and short fourth metacarpals in sisters: A new syndrome? | Presentation of a new osteolytic syndrome | Description of clinical cases. Differential diagnosis of this new syndrome and other osteolytic syndromes. | 2 | Minnesota  (United States of America) |
| Schawo et al. (28)(2006) | Junge frau mit rückenschmerzen und akroosteolysen. Radiologe. | Presentation of a case and to inform on the diagnosis of HCS. | Presentation of a case. Clinical orientation to the diagnosis of HCS is based on physical appearance and radiological findings. | 1 | Heidelberg  (Germany) |
| Albano et al.(64) (2007) | Phenotypic overlap in Melnick-Needles, serpentine fibula-polycystic kidney and Hajdu-Cheney syndromes: A clinical and molecular study in three patients. | To establish the differential diagnosis between Melnick-Needles, HCS and SFPCK. | Differential diagnosis of HCS and the other two syndromes through the description of three cases. Confirmation is obtained of HCS and SFPCK belonging to the same syndrome and that the mutations that cause them are on the same gene. Melnick-Needles, however, is a different disorder as it is cause by a mutation on FLNA. | 3 | São Paulo  (Brasil) |
| Gripp et al.(24) (2011) | Lateral meningocele syndrome and Hajdu-Cheney syndrome: Different disorders with overlapping phenotypes. | To study the differential diagnosis between HCS and lateral meningocele. | Authors perform a comparative study between HCS and lateral meningocele by analysing previously described cases. | - | Wilmington  (United States of America) |
| Gripp et al.(63) (2015) | Truncating mutations in the last exon of *NOTCH*3 cause lateral meningocele syndrome | Genetic and clinical analysis of lateral meningocele syndrome | Description and comparison of five cases and approach to the differential diagnosis with HCS. | 5 | Philadelphia  (United States of America) |
| Damian et al.(62) (2016) | Capillaroscopic findings in a case of Hajdu-Cheney syndrome | To present capilaroscopy ad a diagnostic tool in HCS. | Description of the first case of HCS where capilaroscopic findings are seen. Capilaroscopy as a tool for early diagnosis. | 1 | Cluj-Napoca  (Rumania) |
| *Treatment* | | | | | |
| Liljeström et al. (73)(2003) | Occlusal rehabilitation of a patient with hereditary multicentric osteolysis. | Presentation of a case of HCS. | Description of a case with HCS who required dental restauration. | 1 | Turku  (Finland) |
| Al-Mayouf et al.(68) (2006) | Cyclic intravenous pamidronate treatment in children with nodulosis, arthropathy and osteolysis syndrome | To analyse the use of pamidronate as a treatment for osteolytic syndromes. | Description of seven cases and their response to pamidronate as a pharmacological treatment. | 7 | Riyadh  (Saudi Arabia) |
| McKiernan et al.(29) (2007) | Integrated anti-remodeling and anabolic therapy for the osteoporosis of Hajdu-Cheney syndrome | Presentation of a case of HCS who underwent pharmacological treatment. | Description of a case of HCS who was treated pharmacologically for osteoporosis with an anti-remodelling and anabolic therapy. Results obtained suggest dissociation of bone formation. | 1 | Marshfield  (United States of America) |
| Murtagh et al. (27)(2008) | Spinal reconstruction in Hajdu-Cheney syndrome. | Presentation of a case previously described in 1978 who now receives surgical treatment. | Highlights the importance of surgical treatment in patients with HCS who require spinal reconstruction, an effective although aggressive technique. | 1 | Siracusa  (Italy) |
| McKiernan et al.(70) (2008) | Integrated anti-remodeling and anabolic therapy for the osteoporosis of Hajdu-Cheney syndrome: 2-Year follow-up | Evaluation and surveillance of treatment of osteoporosis initiated two years earlier. | After two years of this treatment, the result found is that biochemical markers of bone turnover present an increase in bone resorption and in osteolysis. | 1 | Marshfield  (United States of America) |
| August et al. (76)(2009) | Anesthesia for a child with Hajdu-Cheney syndrome | To study the use of anesthesia in HCS. Presentation of a case of HCS. | Description of a case of HCS that required surgical treatment and needed anesthesia. | 1 | Sacramento  (United States of America) |
| Vingerhoedt et al.(72) (2010) | Syndrome of Hajdu-Cheney: Three case reports of orofacial interest. | Presentation of three cases of HCS. | Description of three cases of HCS with alterations of the mouth and dentures. | 3 | Leuven  (Belgium) |
| Hwang et al.(69) (2011) | Effect of Zoledronic Acid on Acro-Osteolysis and Osteoporosis in a Patient with Hajdu-Cheney Syndrome | To understand the effect of Zoledronic Acid as a treatment for HCS. | Description of a case of HCS who underwent treatment with Zoledronic Acid. Results show that this anti-resorption therapy may favour osteoporosis but not acroosteolysis. | 1 | Yonsei  (South Korea) |
| Tsinopoulou et al. (67)(2012) | Two-year cyclic infusion of pamidronate improves bone mass density and eliminates risk of fractures in a girl with osteoporosis due to Hajdu-Cheney syndrome | Presentation of a case of HCS. | Description of a case of HCS who received pharmacological treatment with pamidronate. | 1 | Athens  (Grece) |
| Ornetti et al. (26)(2012) | Osteoporotic compression fracture revealing Hajdu-Cheney syndrome. | Presentation of a case who underwent surgical treatment. | Analysis of a case of HCS with medullar collapse and the surgical intervention. | 1 | Dijon  (France) |
| Fujioka et al.(74) (2013) | Proximal translation of the radius following arthroplasty of the distal radioulnar joint in Hajdu-Cheney syndrome | Presentation of a case of HCS. | Description of a case with HCS who underwent surgical treatment. | 1 | Kobe  (Japan) |
| Yamaguchi et al.(75) (2013) | A case report of anesthesia for a child with Hajdu-Cheney syndrome | To study the use of anesthersi in HCS. Presentation of a case of HCS. | Description of a case with HCS who required surgical treatment and needed anesthesia. | 1 | Tomishiro  (Japan) |
| Zietz et al. (77)(2013) | Continuous spinal labor analgesia in a patient with Hajdu-Cheney syndrome | Presentation of a case of HCS. | Description of a case of HCS and the use of post-surgical analgesia for pain management. | 1 | Toronto  (Canada) |
| Mattei et al. (71)(2015) | Surgical challenges in the management of cervical kyphotic deformity in patients with severe osteoporosis: an illustrative case of a patient with Hajdu–Cheney syndrome | Presentation of a case of HCS. | Description of a case of HCS that required surgical intervention due to cervical skeletal alterations. | 1 | Buffalo  (United States of America) |
| Adami et al. (66)(2016) | Hajdu Cheney Syndrome; report of a novel *NOTCH*2 mutation and treatment with denosumab. | Presentation of a case of HCS and response to denosumab as a pharmacological treatment. | Description of a case of HCS treated denosumab, obtaining an increase in bone mineral density but a negative progression of acroosteolysis. | 1 | Verona  (Italy) |
| Sakka et al.(30) (2017) | Bone Structural Characteristics and Response to Bisphosphonate Treatment in Children with Hajdu-Cheney Syndrome | To analyse the bone response in HCS to pharmacological treatment with bisphosphonates. | Description of five cases treated with bisphosphonates. Bone mineral density of the lumbar spine decreased at the beginning of the study and increased in response to treatment, an effect that did not persist after interruption of treatment. | 5 | Birmingham  (United Kingdom) |
| Pittaway et al. (65)(2018) | Bisphosphonate therapy for spinal osteoporosis in Hajdu-Cheney syndrome. | To establish and evaluate the efficacy of bisphosphonate therapy as a pharmacological treatment for HCS. | Despite the increase in bone mineral density after the administration of treatment, the results obtained show that response is variable in each patient and age-dependant | 15 | London  (United Kingdom) |
